# Lateral Meristems Responsible for Secondary Growth of the Monocotyledons: A Survey of the State of the Art

**DOI:** 10.1007/s12229-015-9152-8

**Published:** 2015-04-04

**Authors:** Joanna Jura-Morawiec, Mirela Tulik, Muhammad Iqbal

**Affiliations:** 1 Polish Academy of Sciences Botanical Garden – Centre for Biological Diversity Conservation in Powsin, Prawdziwka 2, 02-973 Warsaw, Poland; 2 Department of Forest Botany, Warsaw University of Life Sciences - WULS, Nowoursynowska 159, 02-776 Warsaw, Poland; 3 Department of Botany, Jamia Hamdard (Hamdard University), Tughlaqabad, New Delhi, 110062 India

**Keywords:** Monocotyledons, Etagen meristem, Monocot cambium, Storied cork

## Abstract

This review highlights key historical works and the recent research on the monocot lateral meristems. It discusses the terminological issues (elucidating the terminological inconsistency found in the literature concerned), origination of secondary meristems, their morphology and characteristic features of the derivative tissues. Also the monocot cambium response to hormonal and gravitational stimuli is discussed. The summarized inputs in the present note are believed to renew interest in this field, which is important for a more comprehensive understanding of the abnormal secondary growth in the monocotyledons.

## Introduction

Occurrence of secondary growth due to the activity of two laterally positioned meristems, viz. the vascular cambium and the phellogen (cork cambium) is a common feature of the gymnosperms and dicotyledons. Among the monocotyledons, secondary growth is not so common and is realized by the activity of peculiarly different meristems. The presence of the secondary vascular system has been recognized within 22 genera only (Rudall, 1995) belonging to the Asparagales (Seberg et al., 2012), whereas a protective tissue of secondary origin has been identified in Zingiberaceae, Bromeliaceae, Commelinaceae and Arecaceae (Schoute, 1902; Krauss, 1949; Tomlinson, 1961).

While progressively more is known about the secondary growth common to most vascular plants, the abnormal secondary thickening of monocots remains understudied. As was pointed out by Carlquist (2012) and earlier workers (Tomlinson & Zimmermann, 1967), the non-monocot angiosperms generally form an easier experimental material. Therefore, we know much more about their anatomy and physiology than of the monocots. The apparent lack of interest in research on the monocot secondary tissues may also be connected possibly with their less commercial significance. The major models for the current research of secondary growth in angiosperms are *Arabidopsis thaliana* and the species of the genus *Populus* (Ursache et al., 2012). The latter is important in the boreal forests and in temperate plantations for the pulp and paper production. Similarly, *Quercus suber*, the main source of commercial cork, is of particular interest with regard to its protective tissue formation (e.g. Ramos et al., 2013). Due to the fact that some of the monocots with secondary growth also supply economically important products (like dragon’s blood) and belong to the vulnerable species, e.g. *Dracaena cinnabari* or *D. draco* (IUCN, 2014) the studies concerning their growth pattern are pivotal to our understanding of the process of their development. Moreover, it may be helpful in practical breeding of species like *D. fragrans* or *D. sanderiana* which are popular ornamental pot plants and have the ability to improve the indoor air quality by removing air pollutants (Wolverton et al., 1989; Treesubsuntorn &Thiravetyan, 2012).

It is known that the meristems responsible for secondary growth in monocot plants differ from the cambia of the gymnospermous and dicotyledonous species. However, except for some preliminary information on their origin and activity, little is known about their structure and behavior. In this review attention is focused on the secondary thickening of stems only although a reference has been made to roots in the case of *Dracaena* (Tomlinson & Zimmermann, 1969). We will discuss in particular (a) the terminological issues, (b) development of secondary meristems, (c) their morphology, (d) the characteristics of derivative tissues, and (e) the response of monocot cambium to hormonal and gravitational stimuli. This summary of information on the secondary growth in monocotyledons can initiate discussion on the issues that have so far been enigmatic.

## Terminology

The elusive nature of the lateral meristems in the monocotyledons has been posing problem with the application of relevant terminology. The literature is fraught with synonyms referring (a) to the meristem producing the secondary vascular tissues, e.g. the thickening ring (Scott & Brebner, 1893), the Etagencambium (Schoute, 1902), the meristematic zone (Arber, 1925), the secondary thickening meristem (Clowes, 1961), the anomalous cambium (Stone, 1970), the vascular cambium (Zimmermann & Tomlinson, 1970, 1972), the accessory cambium (Rastogi, 2009), the cambium-like zone (Beck, 2010) and the monocot cambium (Carlquist, 2012), as well as (b) to the meristem producing secondary protective tissues, e.g. the Etagenmeristem (Philipp, 1923; Tomlinson, 1961), the storied meristem (Fahn, 1967), and the storied phellogen (French & Tomlinson, 1981). However, in the case of secondary protective tissues the meristem itself is rarely distinguished, and normally the term *storied cork* is used, covering both the meristematic cells and their derivatives.

**Fig. 1:**
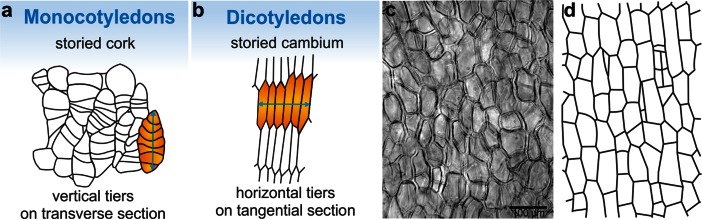
The storied cambium/cork. **a** Storied cork of *Curcuma longa* (Philipp, 1923, modified); **b** Storied cambium of *Laburnum* sp. (Majumdar, 1941, modified); **c** Tangential section of storied cork of *Dracaena draco* stem; **d** Scheme of tangential section of monocot cambium in the stem of *Cordyline terminalis* (Philipson et al., 1971, modified)

The German term *etagen* and its English counterpart *storied* that appear frequently in descriptions of the monocot secondary growth, have been used to underline the temporary form of the monocot meristems. These are zones of cells that do not form a continuous radial file in transverse view due to lack of permanent initials (Schoute, 1902). However, various series, tiers, rows, bands or the so-called stories of cells with only limited radial length become visible in transverse plane (Fig. 1a). It is clear from the above that, currently, one and the same term *etagen/storied* is being applied to two different conditions, depending on the plant group. In the monocotyledons, the term *etagen/storied* relates to the arrangement of cells of the lateral meristem (*etagen* meristem) and the cork tissue (*storied* cork) as visible in transverse plane (Cheadle, 1937; Rudall, 1991; Donoghue, 2005; Evert, 2006; Verma & Khosa, 2012), while in the dicotyledons, it refers to the stratified arrangement of cells of the vascular cambium (Fig. 1b) and the cambium-derived vascular tissues (secondary phloem and secondary xylem) as seen in tangential plane (Bailey, 1923; den Outer, 1986; IAWA, 1989). In the monocotyledons the cork cells do not form tiers in the tangential view (Fig. 1c), likewise the cells of the monocot cambium (Fig. 1d). The discrepancy in the two meanings of the given term is striking. However, both the usages are already well established in the literature and replacement of ‘storied cork’ by a more appropriate term like ‘rowed cork’ may be difficult.

The peculiar monocot cambium produces secondary growth that can be considered as a *true* secondary growth (Fisher, 1973; Fisher et al., 1974; DeMason, 1994) because it is the product of divisional activity of a secondary meristem (Tomlinson & Zimmermann, 1969 and earlier workers).

## The Monocot Vascular Cambium

### Origin of the Meristem

Here, the meristem will be named as secondary thickening meristem (STM) or the monocot cambium, as these two terms most frequently appear in the current literature. The monocot cambium is not to be regarded in any way as a vestige of the initial/vascular cambium that is presumed to have existed in the common lignophyte ancestor of the traditional dicotyledons and monocotyledons (Rudall, 1991; Carlquist, 2012). Ontogenetically, the monocot cambium originates from the primary thickening meristem (PTM), which is a region of actively dividing meristematic cells, located around the apical meristem and extending down the periphery of the stem, where this continuation of PTM is referred to as STM. Thus, the occurrence of PTM is a pre-requisite for differentiation of the STM/monocot cambium (Stevenson & Fisher, 1980; DeMason & Wilson, 1985). According to Diggle and DeMason (1983b), the transition of primary meristem into secondary meristem in the monocotyledonous species is analogous to the transition of procambium to vascular cambium in the woody dicotyledonous stem. On the basis of a detailed study of *Yucca whipplei*, covering the histology (Diggle & DeMason, 1983a, b) and audiography (DeMason & Diggle, 1984) observations, it was concluded that the PTM and the monocot cambium (referred to by them as STM) are ontogenetically related to each other and “function as a single entity during the growth and development of the vegetative stem”. This idea found support from the subsequent observations on *Cordyline terminalis* (DeMason & Wilson, 1985). Earlier, Fahn (1967) also pointed out that if these two meristematic tissues are present in one plant, they could be two developmental phases of the same meristem. Diggle and DeMason (1983a, b) held that the PTM and the STM are histologically similar and are recognizable as a region of radially flattened cells arranged in anticlinal files. A distinction between these meristems was possible usually because of the cell arrangement in derivative tissues, especially those within the vascular bundles. Formation of the amphivasal vascular bundles indicates the presence of the monocot cambium and the commencement of the secondary growth (Diggle & DeMason, 1983b). However, the transitional state is confusing even within the vascular bundles; therefore, when distinction between the PTM and the STM was not possible, the term *thickening meristem* was used (DeMason & Wilson, 1985). Careful structural studies of the monocot cambium could help to check whether additional criteria can be established for a better identification of the secondary meristem.

### Cambial Morphology and Cell Structure

**Fig. 2:**
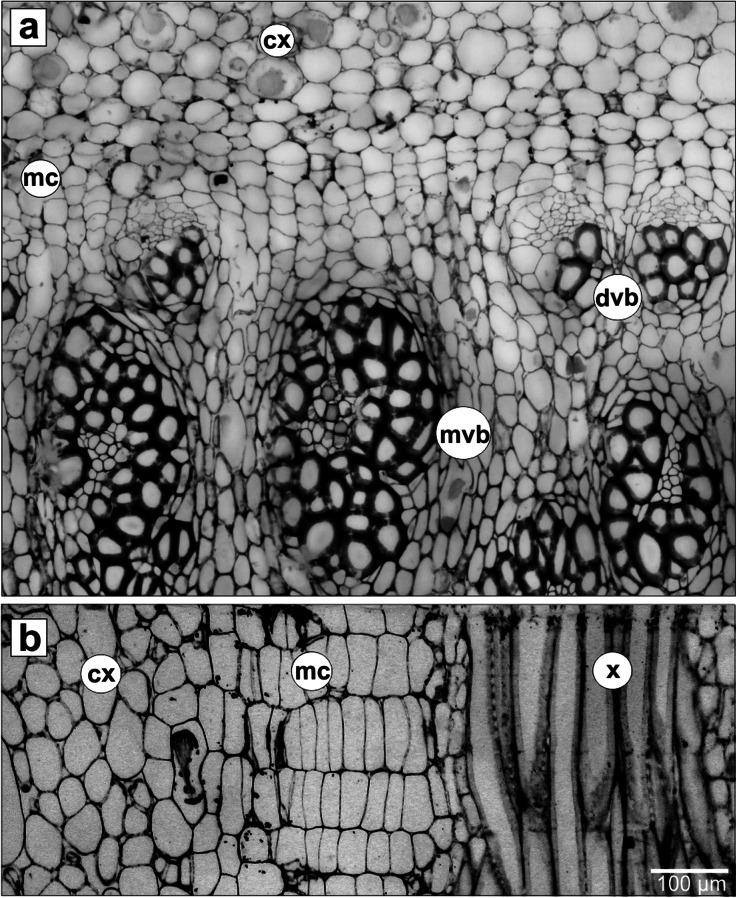
The monocot cambium and its derivative tissues in *Dracaena draco* stem. **a** Transverse and **b** radial sections. *cx* - cortex, *mc* - monocot cambium, *dvb* - developing amphivasal vascular bundle, *mvb* - mature amphivasal vascular bundle, *x* - xylem (tracheids) of the amphivasal bundle

Anatomical studies of the monocot cambium have been only few and the information about its cellular composition is limited. It is known that the monocot vascular cambium is rayless and consists of only one type of cells that look rectangular, fusiform or polygonal in shape. To date, only two photographs, which depict the arrangement of cambial initials in the tangential section, have been published (Cheadle, 1937; Philipson et al., 1971). They present a somewhat nonstoried arrangement of cells (Fig. 1d). In the transverse view, this meristem is described as a multi-layered zone of radially flattened cells, that possess small radial dimension and are tangentially elongated (Fig. 2a). They produce files of derivatives by means of successive periclinal divisions, which is also clearly visible in radial section (Fig. 2b).

Long ago, Cheadle (1937) opined with reference to the monocot cambium that neither an exact location nor a convincing description of the cambial initials was available in the literature, and unfortunately this statement is considerably valid even today. Further, the ultrastructural studies of the monocot meristems seem to have been confined to a single report on *Aphyllanthes monspeliensis*, demonstrating that the active cambial cells are highly vacuolated with protein bodies seen occasionally in the vacuoles (Chakroun & Hébant, 1983).

### Regulation of Cambial Growth

Information about the control of this meristem at the genetic level is, to the best of our knowledge, lacking in the literature. However, some data on the hormonal and gravity induced adjustments of the meristem are available. A preliminary examination of *Cordyline* plants indicated that this was a difficult material for investigating the influence of hormones on the cambial activity because (a) the plants do not form annual growth rings, making it difficult to measure the amount of new accumulation of the secondary tissue produced after the treatment of the meristem with growth regulators, (b) the growth rate of this plant is much slower than one of the dicotyledonous twigs and, therefore, the experiments with growth regulators take much longer and are more prone to the risk of tissue infection (Fisher & Tomlinson, 1972; Fisher, 1973). However, it has been shown that the activity of the meristem responsible for the secondary growth of the monocotyledons is more stimulated by the application of naphthalenacetic acid (NAA) than by that of benzyl adenine (BA) or gibberellic acid (GA) alone (Fisher, 1973). Other experiments carried out with this species revealed that the concentration of auxin in horizontal stems was up to seven times greater in the lower side of the stem axis than in the upper one (Fisher et al., 1974). The information available on biosythesis, transport and signaling of auxin in monocotyledons is based on the research conducted with maize and rice (reviewed by McSteen, 2010). These plants are considered not to be susceptible to auxin during the vascular differentiation, compared with the dicotyledons (Aloni & Plotkin, 1985). Further factors co-acting with auxin are probably required for setting the process in (Scarpella & Meijer, 2004). It is also assumed that the mechanism responsible for auxin metabolism, its movement and transduction is conserved in both monocotyledons and dicotyledons (McSteen, 2010).

**Fig. 3:**
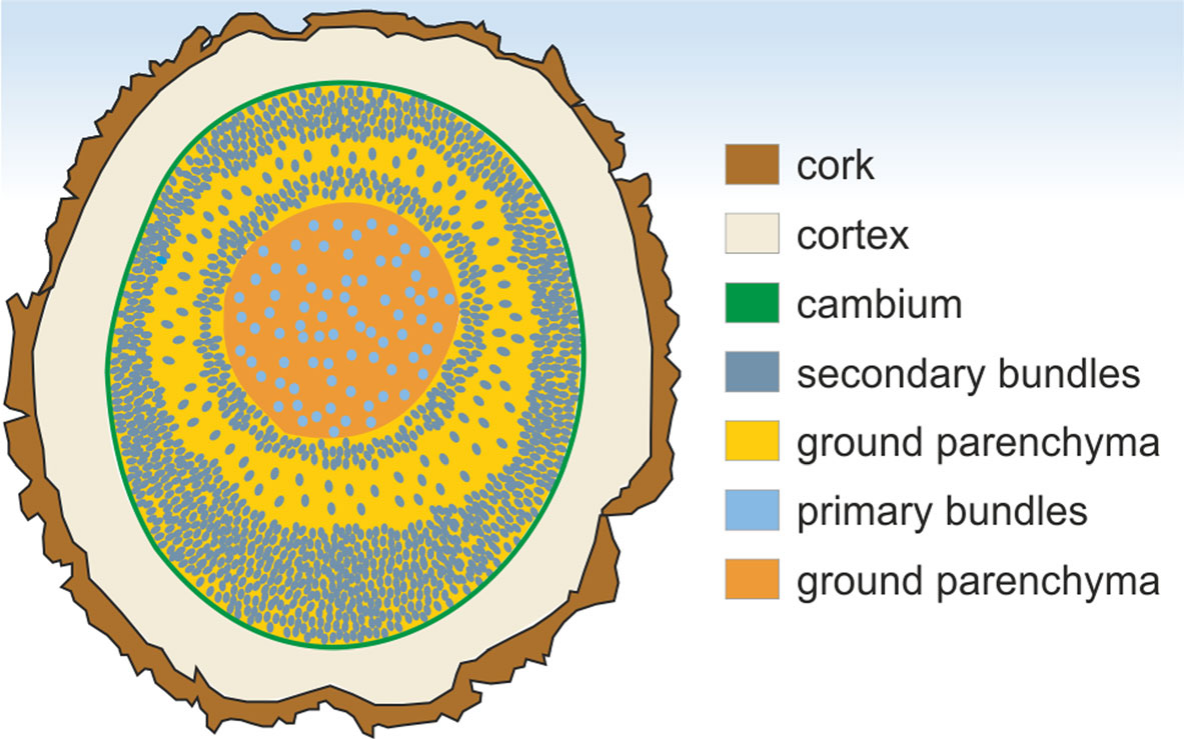
Scheme of the eccentric secondary growth with ‘growth rings’ in stem of *Cordyline terminalis*. The ground parenchyma of the primary and secondary origin is marked with different colors (Jura-Morawiec & Tulik, 2010; modified)

In the horizontal or leaning monocot stems, the monocot cambium produces secondary tissues that are asymmetrically distributed (Fig. 3). As in the conifers, enhanced deposition of secondary tissues takes place on the lower side of the stem, but as opposed to the conifers, without association of the modified tracheids (Tomlinson & Zimmermann, 1969; Fisher, 1975). The lack of anatomical changes characteristic of reaction wood indicates that the eccentric growth in these plants does not result in restoring leaning stem to vertical position, unlike the stem reaction wood in conifers, *Ginkgo* and most of the dicotyledonous plants (possessing compression and tension wood, respectively). This function is probably taken over by the region of primary growth (Tomlinson & Zimmermann, 1969; Fisher, 1975). Then, the significance of the growth eccentricity is rather connected only with stabilization of the dislocated stem, like in *Cycas* with successive cambia (Fisher & Marler, 2006; Altaner et al., 2010). The effect of the leaning position of the stem on the amount of secondary tissues deposited on the lower side is not uniform. Some species, e.g. *Beaucarnea recurvata*, *Y. elephantipes* and *D. reflexa* have shown strong eccentricity of secondary growth, possibly due to intensive cambial activity, in contrast to some others like *C. terminalis* or *D. fragrans* (Fisher, 1975).

The minimum night temperatures, transplanting of plants or the insect infestation may affect the activity of the vascular cambium (Fisher, 1975), resulting in appearance of ring-like structures in the secondary body of monocotyledonous plants (Fig. 3). The alternating ‘dark’ and ‘light’ zones in the secondary body, reported in early studies of monocotyledons (Lindinger, 1909), has been described later in a number of species like *Aloe ferox* (Chamberlain, 1921), *Y. aloifolia* (Barkley, 1924), *B. recurvata* and *C. terminalis* (Fisher, 1975), and *Protoyucca shadishii*, the first reported permineralized monocotyledon with secondary growth (Tidwell & Parker, 1990). The concentric layers of the secondary tissue were referred to as ‘growth rings’ (Cheadle, 1937), as they look like annual growth rings of woody conifers and dicotyledons to the naked eye (Lev-Yadun & Lipschitz, 1986). However, there is no evidence that they correspond to yearly increments. The characteristic alternate zones in the secondary plant body appear due to differences in the size of vascular bundles, relative number of bundles per unit area, wall thickness of parenchyma cells and the size and abundance of parenchyma cells (Cheadle, 1937). In general, the vascular bundles are a little larger and less numerous per unit area, whereas parenchyma cells are unlignified in the ‘light’ zones, in contrast to the bundles and parenchyma in the ‘dark’ zones.

### Cambial Derivatives

The monocot secondary meristem produces most of the derivatives inner to the cambial cylinder with both the secondary phloem and secondary xylem lying on one side, which makes the growth essentially unidirectional (Philipson & Ward, 1965). The parenchymatous secondary conjunctive tissue is deposited both on the internal and external sides; the deposition may be meager as in *Dasylirion serratifolium* or massive as in *Furcraea pubescens* (Schoute, 1903; Cheadle, 1937). The cells on the outer side of the monocot cambium undergo little differentiation; they enlarge about twice the size of the initial cambial cells, become filled with needle-shaped crystals of calcium oxalate, and their walls do not thicken much (Tomlinson & Zimmermann, 1969). More crystals accumulate in the secondary tissue than in the primary one (Lu & Chiang, 1976).

The inner derivatives of the monocot cambium differentiate into cells of secondary conjunctive tissue and vascular bundles. The secondary parenchyma possesses large intercellular spaces, with its component cells often arranged in radial files. The parenchyma cells adjacent to vascular bundles have considerably thickened walls with distinctly visible pits (Barkley, 1924). The other derivatives divide rapidly and differentiate into xylem and phloem cells, which constitute the entire vascular bundles (desmogen strands) (Stevenson & Fisher, 1980). The xylem contained only tracheids with thick walls and circular bordered pits (Carlquist, 2012). These tracheids are about 20 times (Waterhouse, 1987) longer than the cambial cells they derive from. This is largely because of the enormous intrusive growth experienced by the developing tracheids during differentiation. The other cells of the secondary tissue are comparable in length with their initials. The phloem strand is composed of sieve elements and companion cells. Barkley (1924) reported 6–8 sieve tubes with companion cells in the phloem of *Y. aloifolia*, as seen in the transverse section. The constituent cells of the conjunctive tissue may exhibit radial alignment, as does the arrangement of vascular bundles (Stevenson, 1980).

Secondary vascular bundles usually differ from primary bundles in having the amphivasal arrangement of xylem and phloem (xylem surrounding the phloem), whereas the latter usually have a collateral arrangement (Tomlinson & Zimmemann, 1969; Jura-Morawiec & Wiland-Szymańska, 2014). Studies on *Yucca* spp. suggest that the pattern of vascular system is species-specific. In the stem of *Y. aloifolia* the collateral as well as the amphivasal secondary vascular bundles were distinguished (Barkley, 1924). In contrast, these bundles were only amphivasal in *Y. whipplei* (Diggle & DeMason, 1983a, b) and only collateral in *Y. brevifolia* (Carlquist, 2012). It is known that the xylem and phloem patterns within the vascular bundle are subjected to genetic control. The Class III HD-ZIP and KANADI genes, with antagonistic role, are critical for determining the pattern of xylem and phloem within the vascular bundle (Emery et al., 2003). Most research in this field has been done with the dicot model plant *A. thaliana*. However, it is hypothesized that these genes are involved in more general patterning system that appeared early in or prior to the land-plant evolution (Floyd & Bowman, 2007), and hence are good genetic markers for understanding the morphological and developmental innovations achieved during the evolutionary history of land plants. Research on the Class III Homeodomain Leucine Zipper gene family members in rice has partly confirmed that they have conserved functions with their homologs in *A. thaliana* (Itoh et al., 2008). The interaction of the class III HD-ZIP/KANADI genes in the stem of monocots with secondary growth remains unexplained so far, although it has provided an interesting direction for future work (Dinneny & Yanofsky, 2004).

The functional role of the secondary plant body is connected with the mechanical support and the storage of food or water (Cordemoy, 1893; Holm, 1894; Lindinger, 1909). The hard lignified ring of the secondary ground parenchyma may play supportive function (e.g. *Dracaena*), whereas unlignified ground parenchyma takes part in food storage (e.g. *Yucca*). In desert species (e.g. *Beaucarnea*) secondary tissues can maximize the availability of water.

Based on a single report concerning *D. mannii*, it is known that despite exhibiting different anatomical features the monocot secondary tissues possess mechanical properties comparable with those of the dicotyledonous wood of similar density; the difference is confined only to the extent of the radial and tangential shrinkage (Torelli & Trajković, 2003).

## The Secondary Meristem for Protective Tissue Formation

As mentioned above in the section ‘Terminology’, the meristem that gives rise to the secondary protective tissue in monocots is rarely distinguished from derivatives in normal description. Therefore, the general designation of the secondary protective tissue, i.e. *storied cork*, refers to both the meristem and its derivative cells. The presence/absence of this tissue is considered as a diagnostic trait (Tenorio et al., 2012).

**Fig. 4:**
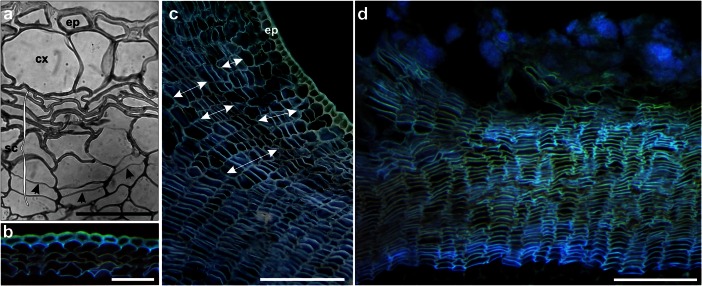
Structural details of *Dracaena* protective tissues: transverse sections from the epon- **a** and wax-embedded **b-d** materials. Bright field and the autofluorescence induced by UV light. **a** *D. draco*; early stages in development of secondary protective tissue, periclinal divisions of meristematic cells were highlighted with arrows, *sc* - storied cork, *ep* - epidermis, *cx* - cortex. **b** *D. marginata,* arrangement of cork cells in young stem. **c, d** *D. fragrans*, storied cork with groups of cells marked with arrows **c**, ‘rhytidome’-like zone **d**. *Scale bars*: a, b 100 μm; c, d 200 μm

In general, meristematic cells arise from some parenchyma cells in the peripheral layers of the cortex (subepidermis) that undergo dedifferentiation and start to divide periclinally (Fig. 4a, b). The meristematic cells are distributed among undivided cortical cells and do not form a continuous layer (Schoute, 1902) characteristic of a typical phellogen (Junikka, 1994; Waisel, 1995). Moreover, these initials have a limited divisional activity (Esau 1965). Thus the arrangement of cells in the *storied cork* might be more or less regular. Krauss (1949) pointed out that sometimes divisions of isolated initials give rise to irregularly placed groups of cells (Fig. 4c). However, in some cases the cell arrangement pattern can be regular, suggestive to the presence of a cork, like one formed by the phellogen in conifers and dicotyledons, when a number of laterally adjacent cells undergo divisions. Contrary to the phellogen of conifers and dicots, the meristem deposits only one type of derivatives i.e. cork cells, the phelloderm is absent. During the course of their differentiation there probably occur the cell wall apposition with suberin and incrustation by lignin, followed by the programmed cell death, suggesting the existence of the same stages of development as in the cork cells of other plants (Krishnamurthy et al., 2000).

In the non-monocot woody plants, the epidermis subjected to pressure due to meristematic activity of vascular cambium is completely replaced by the periderm. The monocotyledons do not develop a type of periderm like that of dicotyledons or conifers (Weisse, 1897; Philipp, 1923). Sometimes, even in older individuals as in *D. fragrans* for instance, both the modified epidermal cells of primary origin and the storied cork are considered to fulfill the role of the protective tissue (Jura-Morawiec & Tulik, 2013). The epidermal cells possess thick and lignified tangential and radial walls as well as the outer tangential walls covered by cutin like-deposition (Fig. 4c).

In the anatomical/botanical sense the rhytidome (successive periderms interspersed with the non-conducting phloem) does not exist in the woody monocots, but the successive layers of the cork are separated by suberized undivided cortical cells (Philipp, 1923). Thus, it makes the whole structure similar to the rhytidome of conifers and dicots in appearance (Fig. 4d).

## Conclusion

The two meristematic tissues responsible for the secondary growth in the monocot species differ from the vascular cambium and the cork cambium of conifers and dicotyledons. The history of research in this field dates back to 19th century; however, these meristems could not draw enough attention of researches, resulting eventually in a lopsided understanding of the monocot secondary meristems in comparison to those typical for most vascular plants. In consequence, we are still stuck with (a) terminological discrepancy regarding the cellular organization of the meristems taking part in the secondary increment, that can be misleading and (b) incomplete basic knowledge gained by using the traditional tools. So far no quantitative studies and critical analyses at the genetic level have been undertaken with reference to secondary growth in the monocot species. This review is believed to stimulate new research on the understudied phenomena of the monocot secondary meristems and their derivatves so as to produce a comprehensive account and a complete picture of the intricacies involved.
